# Acid specific dark quencher QC1 pHLIP for multi-spectral optoacoustic diagnoses of breast cancer

**DOI:** 10.1038/s41598-019-44873-1

**Published:** 2019-06-12

**Authors:** Sheryl Roberts, Arianna Strome, Crystal Choi, Chrysafis Andreou, Susanne Kossatz, Christian Brand, Travis Williams, Michelle Bradbury, Moritz F. Kircher, Yana K. Reshetnyak, Jan Grimm, Jason S. Lewis, Thomas Reiner

**Affiliations:** 1 Department of Radiology, Memorial Sloan Kettering Cancer Center, 1275 York Avenue, New York, New York, 10065 USA; 2 Molecular Pharmacology Program, Sloan Kettering Institute for Cancer Research, New York, New York, 10065 USA; 3 Center for Molecular Imaging and Nanotechnology (CMINT), Memorial Sloan Kettering Cancer Center, New York, NY, 10065 USA; 4 Department of Radiology, Weill Cornell Medical College, 1300 York Avenue, New York, New York 10065 USA; 5 Department of Imaging, Dana-Farber Cancer Institute/Harvard Medical School, Boston, MA 02215 USA; 6 Department of Physics, University of Rhode Island, 2 Lippitt Rd, Kingston, RI 02881 USA; 7 Department of Molecular Pharmacology, Memorial Sloan Kettering Cancer Center, New York, New York, USA; 8 Department of Pharmacology, Weill Cornell Medical College, New York, NY, USA; 9 Molecular Pharmacology Program, Memorial Sloan Kettering Cancer Center, New York, NY USA; 10 Radiochemistry and Molecular Imaging Probes Core, Memorial Sloan Kettering Cancer Center, New York, NY USA; 11 Chemical Biology Program, Memorial Sloan Kettering Cancer Center, New York City, NY 10065 United States

**Keywords:** Diagnostic markers, Sensors and probes, Peptides, Breast cancer

## Abstract

Breast cancer is the most common type of malignant growth in women. Early detection of breast cancer, as well as the identification of possible metastatic spread poses a significant challenge because of the structural and genetic heterogeneity that occurs during the progression of the disease. Currently, mammographies, biopsies and MRI scans are the standard of care techniques used for breast cancer diagnosis, all of which have their individual shortfalls, especially when it comes to discriminating tumors and benign growths. With this in mind, we have developed a non-invasive optoacoustic imaging strategy that targets the acidic environment of breast cancer. A pH low insertion peptide (pHLIP) was conjugated to the dark quencher QC1, yielding a non-fluorescent sonophore with high extinction coefficient in the near infrared that increases signal as a function of increasing amounts of membrane insertion. In an orthotopic murine breast cancer model, pHLIP-targeted optoacoustic imaging allowed us to differentiate between healthy and breast cancer tissues with high signal/noise ratios. *In vivo*, the sonophore QC1-pHLIP could detect malignancies at higher contrast than its fluorescent analog ICG-pHLIP, which was developed for fluorescence-guided surgical applications. PHLIP-type optoacoustic imaging agents in clinical settings are attractive due to their ability to target breast cancer and a wide variety of other malignant growths for diagnostic purposes. Intuitively, these agents could also be used for visualization during surgery.

## Introduction

Malignant growths originate from alterations in the genome, including changes in epigenetic regulation, point mutations, gene deletions, duplications and chromosomal rearrangements^1^. While there have been significant developments in diagnosis and treatment of malignant tumors, treatment of many cancers remains a challenge due to difficulties in diagnosing and identifying them without advanced, and infrastructure-heavy equipment. This is particularly relevant in breast cancer, the most common diagnosed cancer in women, affecting almost 270,000 new individuals yearly in the US alone^2–4^. For breast cancer, early detection of primary tumors significantly improves overall survival^2^. The survival differences are most pronounced between breast cancer patients that were diagnosed with either local versus non-local tumors. Women with localized tumors, which represent 60% of the total breast cancer population, have a >99% 5-year survival rate. The survival rate drops to 70–85% for regional and to 25% for distant metastases. Intuitively, diagnosing breast cancer early represents major benefits in terms of increased survival and also decreased number of treatments. For increasing the number of patients who are diagnosed when their disease is still local, non-invasive and early screening technologies are critical.

Regular systematic screening for breast cancer is therefore key in increasing the overall survival rate^5,6^. Despite improvements in mammography techniques, breast magnetic resonance imaging (MRI) is complementary for screening asymptomatic women at increased risk of breast cancer^7^. This is partly due to the limited sensitivity of mammograms, which declines markedly with increasing breast density and leads to false-negative results^5,8,9^. Women with high breast densities are often screened with ultrasound (US)^10^, but detected abnormalities are often found to be not cancer^11,12^. The combination of these screening tests (mammography/ultrasound and breast MRI) is effective, but it is avoided in practice for an average risk patient due to higher cost^13,14^. Optoacoustic imaging on the other hand has evolved into a clinically translatable platform which can provide limited functional and molecular information^15^. The impact of optoacoustic imaging has been shown through a number of clinical studies for various breast screening of patients^16–20^. It has the primary advantage of imaging at depths of up to 7 cm with submillimeter resolution, high contrast to noise ratio, real time acquisition, portability and does not require ionizing radiation^21,22,23^. In the near future, optoacoustic imaging systems could be the cost-effective alternative for first point of care testing.

So far, optoacoustic imaging often relied on contrast free methods, but to enable a molecularly specific readout, sonophores could be necessary^24^. In breast imaging, tumor heterogeneity limits the general use of cell surface biomarkers for breast cancer imaging^25,26^. On the other hand, one general finding which is largely independent from an individual tumor cell’s expression profile is that the pH at the surface of cancer cells is lower than bulk extracellular pH^27^. The surface acidity of cancer cells is a general physical characteristic that is independent of tumor perfusion and found across an entire tumor^26,28–30^. Therefore, a low pH is a key characteristic in an alternative targeted imaging approach. We hypothesized that a low pH optoacoustic tracer would enable us to non-invasively image breast cancers.

The water soluble pH low insertion peptides (pHLIPs) are a class of membrane-binding peptides which target acidity at the surface of cancer cells^31^. Their mechanism of action is based on the protonation of aspartic and glutamic acid residues, which induces a conformational change of pHLIP’s secondary structure and allows the peptide to insert itself into cell membranes. Indocyanine green (ICG) is a well-established fluorescent dye in scientific literature and its use in humans is known for a variety of fluorescence and optoacoustic imaging applications^32–38^. A pHLIP peptide conjugated with indocyanine green (ICG) has recently been reported to differentiate normal from neoplastic tissue in various animal tumor models^39–42^. To date, however, only one study was carried out using a fluorescent pHLIP for optoacoustic imaging^43^. One of the most striking advantages of ICG-pHLIP is that upon binding to cells, fluorescence is enhanced by 20–25 times, which increases the tumor to background ratio^35^. While, intuitively, this enhancement represents a significant advantage for fluorescence imaging, it is a disadvantage for optoacoustic imaging, a technology that is based on the generation of acoustic signals which rely on the energy transfer to heat (non-radiative decay). An imaging agent which does not disseminate its absorbed energy through photons could therefore be more advantageous to consider for optoacoustic applications.

With this in mind, we became interested in exploring whether dark quenchers could achieve stronger optoacoustic signals than ICG^44–46^. Dark quenchers have gained some attention and holds promise to generate higher optoacoustic signals than their more commonly used fluorescent counterparts since they are strong absorbers and undergo non-radiative decay^44,45,47,48^. The dark quencher IRDye QC1 is structurally similar to ICG (Fig. S1a), but features an aromatized ring system at its center, increasing the rigidity of the structure by reducing internal energy conversion and the tendency for self-aggregation^49^. IRDye QC1 is therefore structurally more robust than ICG.

Using the dark quencher conjugated to pHLIP, QC1-pHLIP, we aim to explore two clinically relevant questions: (i) Does multi-spectral optoacoustic tomography (MSOT) with QC1-pHLIP allow non-invasive delineation of breast cancer in a murine orthotopic breast cancer model? (ii) How does the sonophore QC1-pHLIP compare as an optoacoustic tool to fluorescent ICG-pHLIP? We believe that molecularly targeted sonophores can overcome several hurdles currently hampering the development and broad clinical use of the MSOT. We believe that optimizing and validating QC1 pHLIP for cancer detection is of high value for detecting both localized and non-localized breast tumors. Such an optoacoustic imaging strategy could reduce the frequencies of biopsies, provide accurate monitoring of breast tumors and increase the precision of surgical interventions.

## Results

### Optoacoustic characterization of ICG-pHLIP and QC1-pHLIP

**Figure 1:**
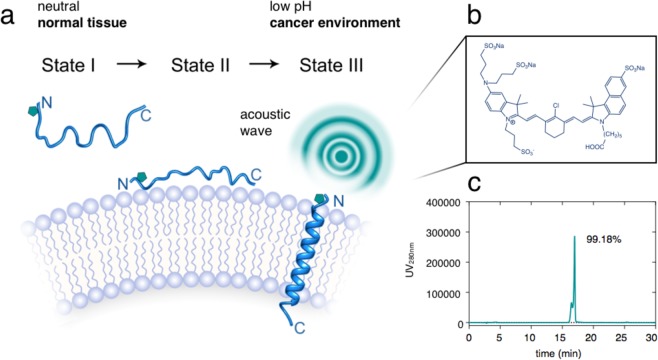
QC1 pH low insertion peptide (pHLIP) targets acidity at the surface of cancer cells and allows multi-spectral optoacoustic imaging. (**a**) Schematic of var3 pHLIP at three distinct conditions (*left to right*). pHLIP is unstructured in aqueous solution at physiological pH (state I) and it binds to the surface of a cell membrane (state II). At acidic pH, pHLIP changes into an α-helix conformation and inserts in between the membrane with the C-terminal found within the intracellular space and the N-terminal extruding towards the extracellular space. (**b**) The structure of IRDye QC1 conjugated to the N-terminal of var3 pHLIP. (**c**) High performance liquid chromatogram (HPLC) at 280 nm of QC1 var3 pHLIP.

**Figure 2:**
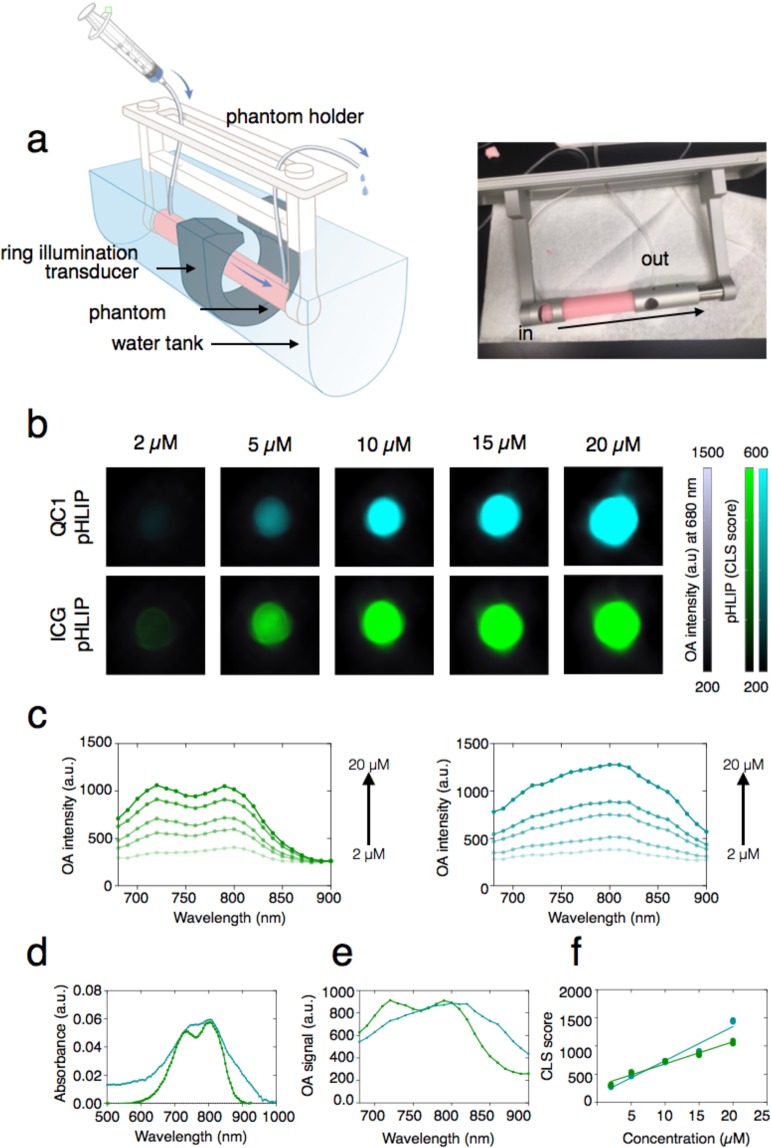
Fluorescent ICG-pHLIP and dark quencher QC1-pHLIP have comparable optoacoustic intensities at the same concentration in tissue mimicking phantoms. (**a**) An optoacoustic imaging flow setup using soft-tissue mimicking phantoms (*right*) was used with the MSOT whole animal imaging setup (*left*). (**b**) Optoacoustic characterizations of ICG-pHLIP and QC1-pHLIP at varying concentrations (2 µM, 5 µM, 10 µM, 15 µM and 20 µM) in DMSO. Optoacoustic intensities at 680 nm were overlaid with the spectrally unmixed signals of ICG-pHLIP (green) or QC1-pHLIP (cyan). (**c**) The optoacoustic spectra (a.u.) of ICG-pHLIP (*left*) and QC1-pHLIP (*right*). (**d**) The UV/Vis absorbance spectra of ICG-pHLIP and QC1-pHLIP at 5 µM deviates from the optoacoustic spectra of ICG-pHLIP and QC1-pHLIP (**e**, *middle*) taken at the same concentrations (15 µM). (**f**, *right*) Classical least square (CLS) fit at varying concentrations of the two pHLIPs after multi-spectral unmixing with the spectra references shown in (**e**, *middle*).

Variant3 pH low insertion peptide (pHLIP) is a synthetic peptide derived from the C-helix of bacteriorhodopsin which contains 27 amino acids with the following sequence: (D-Ala)-(D-Cys)-(D-Asp)-(D-Asp)-(D-Gln)-(D-Asn)-(D-Pro)-(D-Trp)-(D-Arg)-(D-Ala)-(D-Tyr)-(D-Leu)(D-Asp)-(D-Leu)-(D-Leu)-(D-Phe)-(D-Pro)-(D-Thr)-(D-Asp)-(D-Thr)-(D-Leu)-(D-Leu)-(D-Leu)-(D-Asp)-(D-Leu)-(D-Leu)-(D-Trp). To provide the first optoacoustic dark quencher pHLIP, the focus was on obtaining QC1-pHLIP as an acid specific probe, capable of sensing and targeting acidity at the surface of cancer cells in tumors. The scheme of QC1-pHLIP and its mode of action is shown in Fig. 1. To obtain the dark quencher, IRDye QC1 maleimide was cross-linked to a pH sensitive var3 pHLIP at the second amino acid position which is a cysteine (-SH) to obtain QC1-pHLIP (70%). Both IRDye QC1 (Fig. S1a (*left*)) and ICG (Fig. S1a (*right*)) belong to the cyanine dye family. The IRDye QC1 is a subclass of heptamethine type, containing a heterocyclic ring in the center giving rise to a more photostable dye than its parent cyanine family dyes. Full chemical characterization of QC1-pHLIP is shown in Fig. S1. At the same HPLC buffer conditions, the retention time, t_R_ of var3 pHLIP was found at 17.9 min (95.3% AcN), whereas QC1-pHLIP was found at 17.0 min (99.2% AcN). The retention time of ICG-pHLIP was 28.0 min (97.7% AcN). At the same concentration, the appearance of QC1-pHLIP is dark teal, whereas ICG-pHLIP appears green. The extinction coefficient of QC1-pHLIP at 810 nm is 41,000 cm^−1^ M^−1^ and it is similar to the extinction coefficient of ICG-pHLIP at 810 nm which is 42 000 cm^−1^ M^−1^, shown in Fig. S2. Optoacoustic characterizations were assessed using a custom-made optoacoustic flow phantom device that was built to fit into the commercially available MSOT phantom holder (Fig. 2a). Described in the methods section in detail, a phantom was prepared to mimic the light absorption, scattering and depth attenuation of soft tissue. Briefly, the agarose phantom contained Direct Red 81 to mimic the optical absorption and lipids to mimic the scattering of light. Increasing the amount of agarose from 1.5% to 5% permitted a more robust imaging flow set up. Using this setup, the optoacoustic signals of ICG-pHLIP and QC1-pHLIP were tested at various concentrations of 2 µM, 5 µM, 10 µM, 15 µM and 20 µM (all groups n = 3) and were found to be comparable to each other in the absence of cells (Fig. 2b). The corresponding optoacoustic spectra (a.u.) of ICG-pHLIP (Fig. 2c, *left*) and QC1-pHLIP (Fig. 2c, *right*) are shown and directly compared with the shape of its UV/Vis absorption spectra (Fig. 2d). ICG-pHLIP has a distinct optoacoustic shoulder band at 710 nm, whereas QC1-pHLIP notably only has one broad peak (Fig. 2e). A classical least square (CLS) model was used for spectral unmixing of signals, shown in Fig. 2f, using the optoacoustic spectra found in Fig. 2e.

### Concentration dependent increase of ICG-pHLIP loses optoacoustic signal *in vitro*

**Figure 3:**
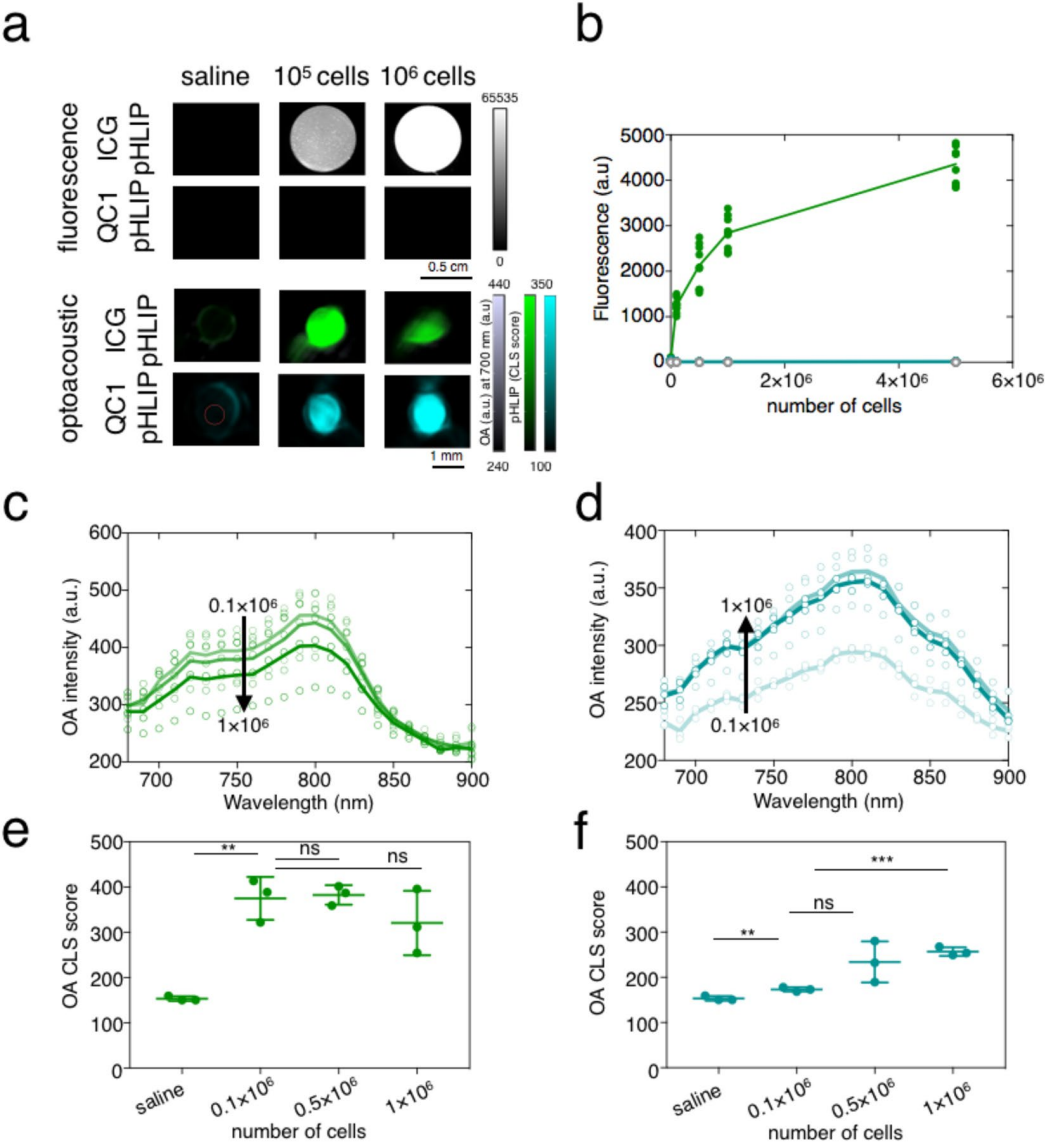
*In vitro* fluorescence and opposing optoacoustic binding signal of fluorescent ICG-pHLIP (green) and dark quencher QC1-pHLIP (cyan) to murine breast cancer cell line 4T1. (**a**) ICG-pHLIP (5 µM) is a turn-on fluorescent probe with zero fluorescence in cell-free media. Fluorescence was immediately observed when pHLIP inserts into the lipid bilayer of plasma membrane at low pH found at the surface of 4T1 cells (*n* = 9, scale bar from 0 to 65535). Optoacoustic signal is inversely proportional to the number of cells incubated with ICG-pHLIP, whereas it increases proportionally for cells incubated with QC1 pHIP. (**b**) Quantification of ICG-pHLIP fluorescence signal increases as the number of cells increases. QC1-pHLIP has negligible signal. (**c**) For ICG-pHLIP, overall optoacoustic instensities (a.u) decreases as the number of cells incubated with ICG-pHLIP (5 µM) is increased. (**d**) For QC1-pHLIP, optoacoustic signal increases as the number of cells incubated with QC1-pHLIP (5 µM) is increased. (**e**) Spectrally unmixed signal shows ICG-pHLIP is a signal-off optoacoustic agent and complicates quantification, whereas (**f**), QC1-pHLIP is a signal-on probe, provides clear quantifiable signal with high significance. Ns = not significant, *p < 0.05, **p < 0.01, ***p < 0.001, ****p < 0.0001 (unpaired t-test).

Fluorescent dyes such as ICG are known to quench at higher concentrations^50^. While evaluating the acoustic nature of the pHLIP imaging agents *in vitro*, the fluorescent behavior of ICG-pHLIP was also assessed. ICG-pHLIP was incubated with increasing numbers of cells of the murine breast cancer cell line 4T1 (0.1 × 10^6^, 0.5 × 10^6^, 1 × 10^6^ and 5 × 10^6^ cells) in phosphate buffered saline (PBS) media containing 10% glucose for 7 min at 37 °C). Increasing the concentrations of 4T1 cells while keeping the pHLIPs concentration constant at 5 µM, yielded dramatically increased fluorescence (Fig. 3a,b). The biggest signal change occurs when changing from cell-free media to 0.1 × 10^6^ cells (16-fold increase). Fluorescence signal difference starts to plateau after that (Fig. S6) with only 30%–70% signal enhancement going from 0.1 × 10^6^ to 5 × 10^6^ cells. As expected and shown in Fig. 3a, no fluorescence was observed for QC1-pHLIP *in vitro* (Fig. S7). For ICG-pHLIP, binding to cells causes a dramatic increase in fluorescence (Fig. 3a (*top*) and 3b), and it was therefore critical to assess the behavior of both the fluorescent ICG-pHLIP and dark quencher QC1-pHLIP in an optoacoustic *in vitro* setting. Using the same experimental conditions carried out in the fluorescence imaging setting, the optoacoustic flow phantom device was used. Plotting the optoacoustic imaging intensities (a.u.) at various wavelengths (680–900 nm, n = 3) and spectrally unmixed CLS score for ICG-pHLIP (Fig. 3c,e), showed that there was a trend that the signal was decreasing with increasing concentrations of 4T1 cells (optoacoustic intensities p > 0.05, CLS score p > 0.05). In comparison, the optoacoustic signal of QC1-pHLIP was found to increase with increasing concentrations of 4T1 cells (Fig. 3d,f, *n* = 3) and the statistical significance was found between cell-free media and 0.1 × 10^6^ cells (****p = 0.0001) and 1 × 10^6^ cells (optoacoustic intensities ****p < 0.0001, CLS score ***p = 0.0002).

### Dark Quencher QC1-pHLIP outperforms fluorescent ICG-pHLIP in *ex vivo* optoacoustic imaging

**Figure 4:**
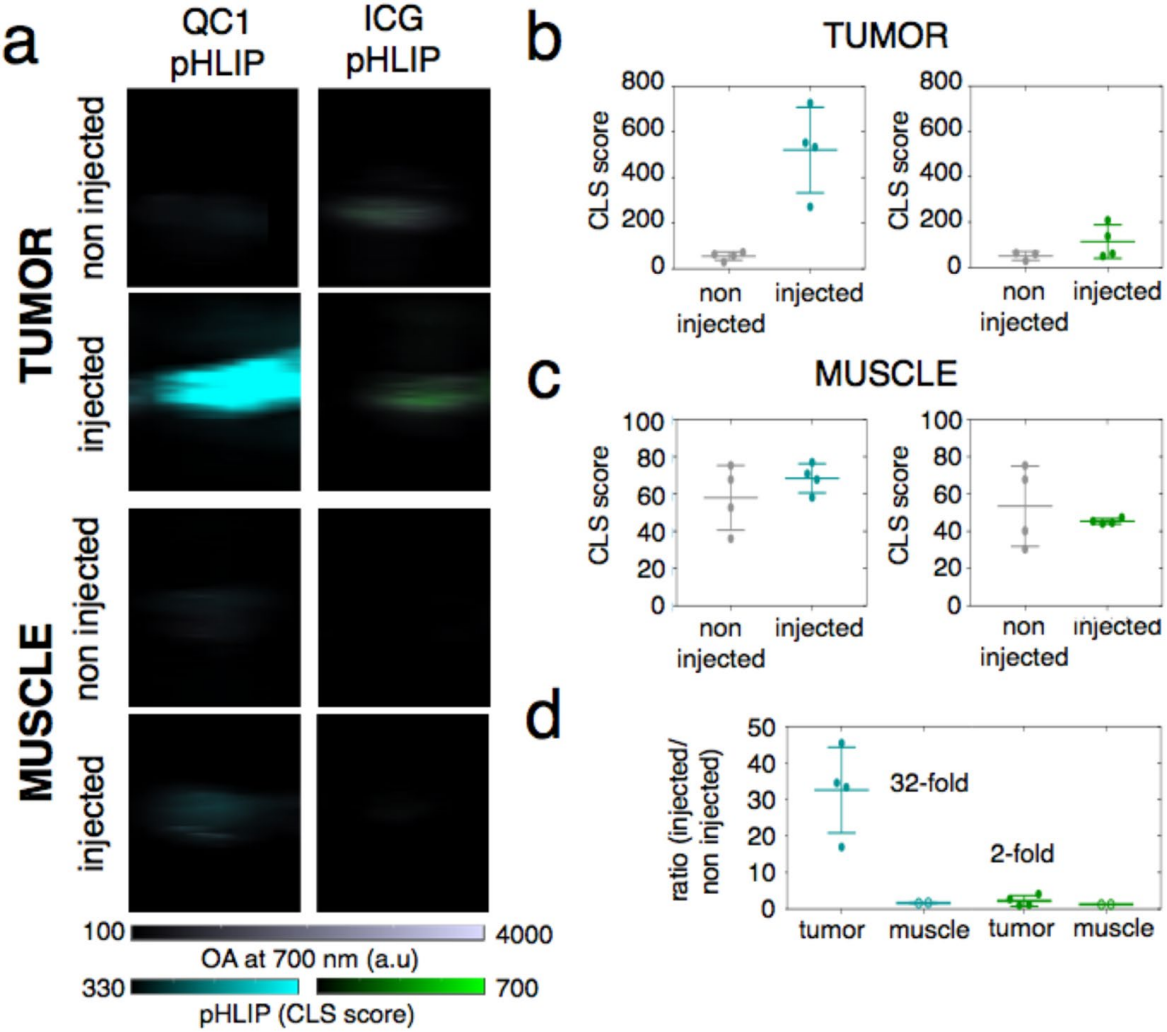
Dark quencher QC1-pHLIP outperforms fluorescent ICG-pHLIP in *ex vivo* optoacoustic imaging 24 h post intravenous injection in an orthotopic murine breast cancer model. (**a**) Optoacoustic images of tumors (*top*) and muscles (*bottom*) harvested from mice, non injected or injected with either QC1-pHLIP (cyan, *left*) or ICG-pHLIP (green, *right*) 24 h post injection. (**b**) Signal from QC1-pHLIP and ICG-pHLIP shows accumulation of the agents in the tumor. (**c**) Muscle tissue was used as control and showed no signal difference between non-injected and injected mice with either agent. (**d**) At the same injected dose of 120 µM, the accumulation of QC1-pHLIP in tumors is more visible than ICG-pHLIP (24 h post intravenous injection), yielding a 33-fold increase in optoacoustic signal as compared to 2-fold increase observed in ICG-pHLIP (each group, *n* = 4 non injected, *n* = 4 injected). Ns = not significant, *p < 0.05, **p < 0.01, ***p < 0.001, ****p < 0.0001 (unpaired t-test).

For comparing the sonophore QC1-pHLIP to its fluorescent counterpart ICG-pHLIP, an orthotopic murine breast cancer model was set up. Imaging for both ICG-pHLIP and QC1-pHLIP (120 µM, 200 µL) was performed 24 h post i.v. injection. The mice were sacrificed and transcardial perfusion was carried out with saline before major organs of interest (tumor, muscle, spleen kidney and liver) were resected. The high optical density of blood represents a major component of the endogenous optoacoustic signal and thus, excised organs were imaged with and without prior perfusion (Fig. S8). Optoacoustic imaging of *ex vivo* organs (ICG-pHLIP, QC1-pHLIP and non-injected controls) were lined up on top of a clear plastic membrane coated with colorless ultrasound gel (Fig. 4a). Optoacoustic signal quantification of tumor (Fig. 4b) and muscle (Fig. 4c) before and 24 h post injection of either QC1-pHLIP (*left*) and ICG pHLIP (*right*) were obtained. *Ex vivo* tumors of QC1-pHLIP injected mice showed higher optoacoustic signals, with QC1-pHLIP showing about 33-fold (mean 32.59 ± 11.74, n = 4) signal enhancement between the injected and non-injected group tumors (**p = 0.003), shown in Fig. 4d. In comparison, ICG-pHLIP injected mice yielded signal enhancement at roughly 2-fold (2.25 ± 1.46, n = 4) and it was found to be not statistically significant (p > 0.05). Muscle tissue was harvested as a control from the same respective mice with ICG-pHLIP (1.25 ± 0.01, n = 4) and QC1-pHLIP 1.67 (1.67 ± 0.10, n = 4). Both of the tracers also accumulated in the spleen, kidney and liver (Figs S9–S10), consistent with earlier reports using labeled pHLIP constructs^40,51–53^.

### QC1-pHLIP yields higher optoacoustic signals *in vivo*

**Figure 5:**
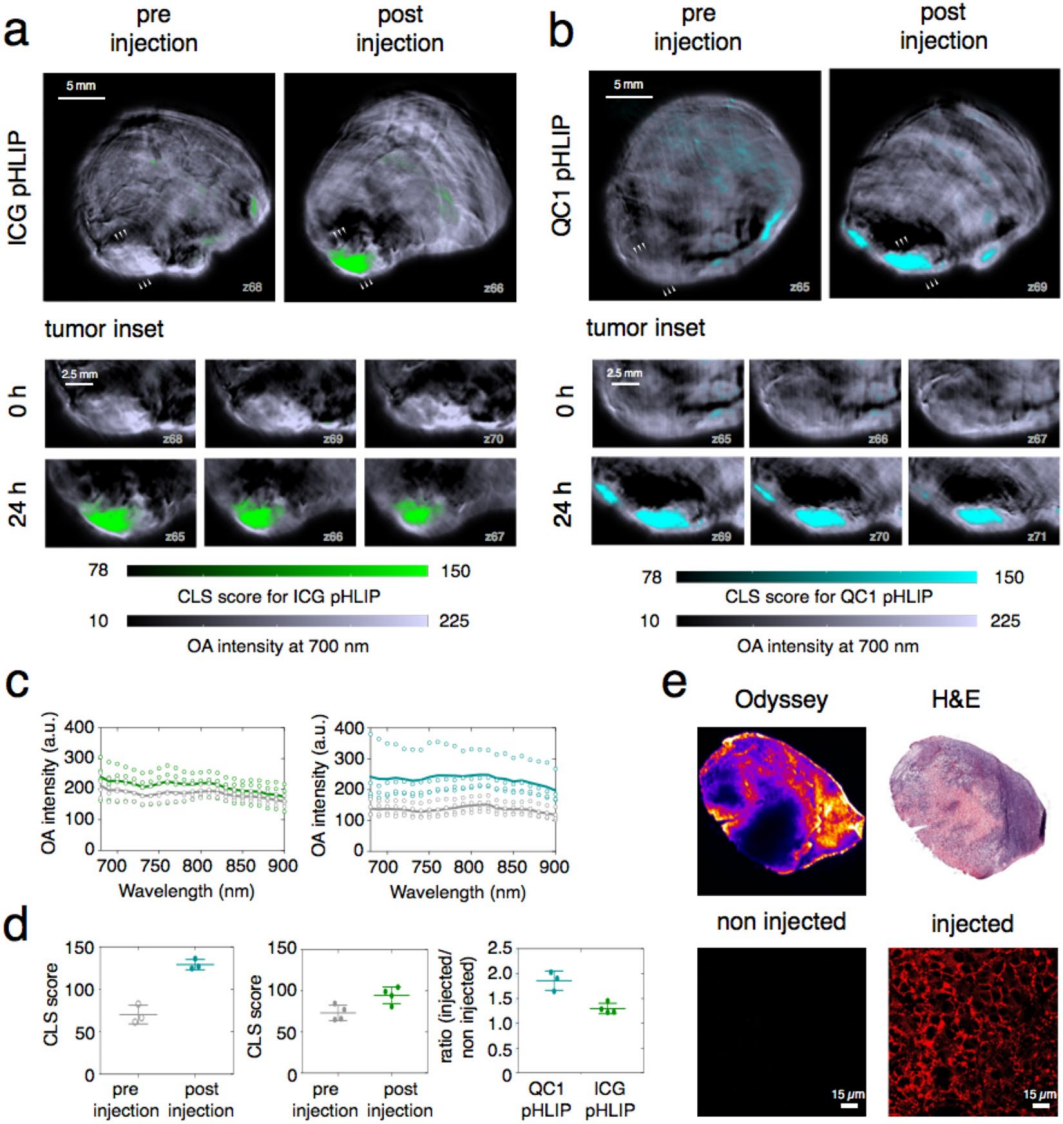
MSOT Imaging performance of dark quencher QC1-pHLIP in a murine breast cancer model outperforms fluorescent ICG-pHLIP. Axial tomographic slices through the tumors before and 24 h after tail vein injection demonstrate specific ICG-pHLIP (**a**) and QC1-pHLIP (**b**) signals in an orthotopic murine breast cancer model. Tomographic sections of tumors from three different planes (xy, xz and yz) demonstrate targeted distribution of both pHLIPs through the tumor. Scale bar (white) is 2.5 mm, optoacoustic intensity (a.u.) scale is from 4.3 × 10^1^ to 2.8 × 10^2^ (grey scale), CLS score scale is from 7.8 × 10^1^ to 2.1 × 10^2^ (green/cyan). (**c**) Overall optoacoustic intensities of pre-injected (grey, n = 4) and post injected mice (*n* = 4) of either ICG-pHLIP (*left*, green) or QC1-pHLIP (*right*, cyan) at varying wavelengths (680–900 nm). (**d**) Representative fluorescent image of excised tumors from ICG-pHLIP injected mice and their corresponding H&E staining. Fluorescent microscopy of the tumors shows membrane insertion and distribution within tumor tissue. No fluorescence was observed in non-injected mice. (**e**) The accumulation of QC1-pHLIP in the tumor yielded 96% signal enhancement as compared to 35% for ICG-pHLIP.

Although ICG-pHLIP and QC1-pHLIP are both highly tumor-specific tracers, the differences in *ex vivo* studies were notable, with QC1-pHLIP clearly outperforming ICG-pHLIP in optoacoustic sensitivity (Fig. 4). Next, the non-invasive MSOT system was used to compare the degree of signal (sensitivity) at the tumor site between ICG-pHLIP and QC1-pHLIP *in vivo*. Representative optoacoustic images of ICG-pHLIP and QC1-pHLIP shown in Fig. 5a,b respectively, showing tumor uptake for both imaging agents. The corresponding tumor insets showing 3 mm of cross-sections shows the accumulation and distribution of the pHLIP agents through the tumor site. Overall optoacoustic intensities, shown in Fig. 5c, for ICG-pHLIP (****p < 0.0001) and QC1-pHLIP (****p < 0.0001) show statistical significant differences between the pre-injected and injected groups at all wavelengths from 680–900 nm. In line with the optoacoustic signal quantification data presented in Fig. 5d, both ICG-pHLIP (**p = 0.0211) and QC1-pHLIP (**p = 0.0013) corroborated statistically significant differences in tumor signal before and after injection (Fig. 5c,d). The background (non tumor regions) produces insignificant optoacoustic signal compared to injected tumor area and it is comparable to the optoacoustic background of the pre injected tumor (Fig. S15). The deoxyhemoglobin (Hb) status of the tumor is useful to locate the tumor margins (Fig. S16) and aid accurate determination for the regions of interest (ROI). QC1-pHLIP yielded a 96% signal enhancement after 24 h post injection, whereas ICG-pHLIP yielded 35% signal enhancement. The fluorescence and histology (H&E) sections showed no signs of toxicity at 120 µM (200 µL) injected dose and the pHLIP-type imaging agent targets the viable tumor tissue of the mouse breast cancer model via membrane insertion (Fig. 5e). The ideal *in vivo* optoacoustic imaging time points for the highest signal/noise ratio was determined at 24 h post injection of QC1 pHLIP (Figs S11 and S12). Further validation were carried out with *in vivo* fluorescence imaging of ICG-pHLIP (Fig. S13). Representative fluorescent images of ICG-pHLIP, QC1-pHLIP and non-injected mice are shown in Fig. S13a at 24 h post injection. It confirms the accumulation of ICG-pHLIP in the tumor and similar pharmacokinetic profile was found for QC1-pHLIP. The accumulation of ICG-pHLIP and QC1-pHLIP starts to plateau between 4 h and 24 h (Figs S11 and S13b).

## Discussion

Although QC1-pHLIP and ICG-pHLIP have similar optoacoustic intensities in the absence of biological materials (Fig. 2), the differences between the two pHLIP constructs became apparent *in vitro and in vivo* (Fig. 3 and Fig. 5). In an orthotopic murine breast cancer 4T1 model that mimics the physiopathology of breast cancer, we tested (i) a pHLIP sonophore and (ii) a pHLIP fluorophore. In 4T1 bearing mice, both agents targeted the acidic environment of breast cancer *ex vivo* and *in vivo* (Figs 4 and 5). The direct comparison of QC1-pHLIP to ICG-pHLIP demonstrated that the dark quencher sonophore yielded higher signal/noise ratios for differentiating malignant from healthy tissues in optoacoustic imaging as compared to the more commonly used fluorophore.

Despite the acidic nature of cancers being known for over 100 years^54^, only in recent decades that acidity has been identified and exploited as a biomarker. Var3 pHLIP was chosen based on its high potential for clinical translation^39^. Not only is var3 pHLIP an effective pH specific probe, differentiating between metastatic lesions and measuring tumor aggressiveness^35^, it has been shown to target several malignant growths that are otherwise hard to diagnose, including bladder cancer^35^, triple negative breast cancers^30^, head and neck cancers^55^ and pancreatic cancers^42,43^. Its unique capabilities make it a potential candidate for universal targeting of cancer, and some pHLIP agents will be entering the clinical realm in 2019^53,56^.

Using an imaging agent that does not emit energy in the form of photons (as fluorophores do) could provide higher signal intensities for optoacoustic imaging applications. QC1, a dark quencher, dissipates its absorbed energy mostly in the form of non-radiative decay. A large portion of energy is emitted *via* internal conversions, which are non-radiative transitions in the form of heat. In addition, QC1-pHLIP targets a low pH environment, a universal biomarker for cancers and offers several advantages for *in vivo* delivery as compared to other contrast agents which have been reported for optoacoustic imaging^57^. Most contrast agents target receptors at the surface of cancer cells, a strategy with several potential drawbacks, including limited agent delivery to the site of the target and an insufficient amount of receptors at the surface to create high signals. Recent fluorescence-based studies of pHLIP show that the substance-class is non-toxic and highly specific to the acidic environment. With this in mind, QC1-pHLIP could be used in targeting a wide variety of cancers in pre-operative imaging where it is essential to determine resectability, particularly with borderline tumors. Due to their size, small peptide based sonophores such as QC1-pHLIP offer better pharmacokinetic and pharmacodynamic properties than non-targeted agents such as nanoparticles that partially accumulate through the enhanced permeability and retention effect (EPR).

The synthesis of the QC1-pHLIP was achieved in one step. The sulfhydryl of single cysteine residue of pHLIP reacts with QC1 maleimide to QC1-pHLIP in high yields (70%). It is important to note that under HPLC neutral buffer conditions where acetonitrile is the mobile phase, two close peaks corresponding to two interchangeable conformations of QC1-pHLIP were observed (Fig. 1c). Attaching a sonophore did not change the ability of pHLIP to protonate under acidic environments. This suggested that the binding of pHLIP was still specific as shown in several biological studies. Several fluorescence and PET pHLIP tracers are undergoing clinical validation^58–60^. Based on this and our data, we believe that pHLIP-based optoacoustic imaging could enter the clinic as well.

ICG-pHLIP has a higher tendency to aggregate and molecularly stack as compared to QC1-pHLIP, which becomes evident at higher concentrations (Fig. 2c). QC1-pHLIP, conversely, is more soluble in physiological conditions owing to the presence of four sulfonate groups and it shows one broad absorbance peak at 810 nm (Fig. 2c (*right*) and Fig. 2d). QC1-pHLIP yields a concentration dependent increase in optoacoustic signal in both phantom (Fig. 2) and *in vitro* studies (Fig. 3d,f). The *ex vivo* biodistribution studies of both ICG pHLIP and QC1 pHLIP show tumor targeting. At the same concentration *i*.*v*. injected (120 µM), QC1 pHLIP shows a higher signal to noise ratio of 32-fold compared to 2-fold with ICG pHLIP. *In vivo*, the signal enhancement for QC1 pHLIP is 100% and 35% for ICG pHLIP. As expected, the presence of endogenous contrast agents at greater depths in the presence of skin account for the dissimilarity between *ex vivo* and *in vivo* results.

Intuitively, it would be premature to assume that sonophores generally make better optoacoustic imaging agents than fluorophores. However, because dark quenchers efficiently absorb energy and do not emit photons, a higher fraction of the absorbed energy could potentially be dissipated as heat, which can make them more potent optoacoustic imaging agents. Our *in vivo* studies corroborate this hypothesis. This suggests that further investigations into the use of dark quenchers as sonophores are not only necessary, but could yield highly significant results. In theory, pHLIP could be conjugated to any other dark quencher. With the current trend pointing towards optoacoustic imaging taking on more and more complex imaging tasks, we believe that studying and understanding the photophysical and biological properties of small molecule sonophores will enable us to create new, better and more sensitive imaging probes for optoacoustic imaging. The systematic chemical studies of these sonophores will potentially increase the likelihood of molecular optoacoustic imaging to become a standard of care technology.

In conclusion, emerging concepts such as pHLIP could provide the discoveries and platforms for new and alternative sonophores in which we can leverage unparalleled capabilities of optoacoustic technologies for cancer detection in patients. Our ability to reverse engineer fluorescent ICG-pHLIP into a dark quencher QC1-pHLIP suggests that routine non-invasive imaging of breast cancer could be achieved with optoacoustically labeled pHLIP sonophore.

## Materials and Methods

### General

*In vacuo* refers to the use of a Büchi Rotavapor R-300 rotary evaporator using the vacuum pump V-710. All reactions were magnetically stirred, and room temperature refers to 20–25 °C. High performance liquid chromatography (HPLC) purification and analysis was performed on a Shimadzu UFLC system equipped with a DGU-20A degasser, SPD-M20A UV detector, a LC-20AB pump system, and a CBM-20A communication BUS module. HPLC solvents (Buffer A: 0.1% TFA in water, Buffer B: 0.1% TFA in CH_3_CN). HPLC purification was performed on an Atlantis dC18 reversed-phase 5 µm silica, 4.6 mm × 250 mm column. Purification was performed using 10–60% CH_3_CN method (gradient: 0–15 min 10–60%, 14 min 60%, 1 min 60-5%) at a flow rate of 1 mL min^−1^. Liquid chromatography-mass spectrometry (LC-MS) using electrospray ionization (ESI) was recorded using a Waters instrument with SQD detector for mass identification. A lyophilizer (FreeZone 2.5 Plus, Labconco, Kansas City, MO, USA) was used for freeze drying. A 100-US high pressure (New Era pump systems Inc., NY, USA) syringe pump was used to control the flow rate and facilitate delivery of solutions and cells inside the phantom flow system. An automated cell counter (Beckman Coulter, Vi-Cell viability analyzer) was used for counting the number of cells. Planar (2-D) fluorescence imaging was carried out using the IVIS Spectrum (PerkinElmer, Waltham, MA, USA). *In vitro* fluorescence confocal microscopy was carried out using a Leica TCS SP8. Fluorescence imaging was also carried out using the Odyssey Classic fluorescence scanner (Li-COR).

### Chemicals

IRDye QC1 maleimide was purchased from LI-COR (Lincoln, NE, USA). Variant 3 (Var3) pHLIP with the amino acid sequence shown in Fig. 1b was purchased from CS Bio (Menlo Park, California, USA). Var3 ICG-pHLIP was provided by pHLIP Inc. (Kingston, RI, USA). Intralipid® 20%, I.V. fat emulsion, Direct Red 81 and agarose Type 1 were purchased from Sigma-Aldrich. All reagents were used without further purification.

### Synthesis of QC1-pHLIP

Var3 pHLIP (2 mg, 0.65 µmol, 1.0 equiv.) was dissolved under nitrogen in DMSO/PBS mixture (300 µL: 700 µL) followed by the addition of IRDyeQC1 maleimide (767 µg, 0.68 µmol, 1.1 equiv.). The reaction mixture was stirred for 24 h at room temperature. The mixture was lyophilized overnight and the dried powder slowly re-suspended in acetonitrile (700 µL). The mixture was purified on a HPLC. The purified product was lyophilized overnight to obtain 360 µg (70% yield) of a teal powder. LC-ESI-MS (ES^+^), *m/z* calculated for [C_202_H_281_ClN_38_O_57_S_5_K_2_]^3+^ 1476.60 found 1475.91 [M + 2 K + H]^3+^. HPLC, t_R_ = 17.0 min (99.2% AcN), λ_max_ (PBS/DMSO) 810/800 nm.

### Chemical characterizations of pHLIPs

Absorption and fluorescence spectra were measured in a 96-well plate (Corning^TM^ Costar^TM^ black clear bottom, Thermo Fisher Scientific) with path lengths of 0.231 cm and 0.300 cm for 75 µL and 100 µL, respectively. UV/Vis absorbance and fluorescence spectra were measured on SpectraMax® M5 Multi- Mode Microplate Reader. Samples were measured together with a corresponding reference solvent contained in a matched well and volume. Measurements were recorded in triplicates at 25 °C. The absorbance scan was performed with an integration time of 0.5 seconds and range from 350 nm to 1000 nm in 5 nm resolution. Spectra and linear calibrations were plotted using Prism 7 (GraphPad Software, La Jolla, CA, USA).

### Tissue mimicking phantom preparation and experimental setup

A custom-made flow-mediated optoacoustic set up was prepared for optoacoustic characterizations of the agents. A clear, cylindrical hollow membrane tubing was placed at the center of a cylindrical mold (20 mL syringe, diameter: 2 cm, length: 14 cm). The soft tissue mimicking phantom was freshly prepared by adding 15% v/v intralipid® 20%, I.V. fat emulsion (to provide the scattering), and 0.01 mM Direct Red 81 (for absorption) to a pre-warmed solution of 5% v/v agarose Type 1 (solid in <37 °C) in Milli Q water (18.2 ΩM cm at 25 °C). Compared to the previously described method^44^, the amount of agarose was increased from 1.5% to 5% v/v, allowing suspension of the phantom in water and supporting the weight of the hollow tubing. The mixture was poured into the mold and allowed to cool and solidify over a minimum of 2 hours. At each end of the tubing, a luer-lock was fitted to connect the syringe pump. The syringe was replaced with a 20 mL syringe filled with PBS. Air gaps were used to separate the sample and PBS. The flow rate was kept at 1 mL min^−1^ until the sample of interest reached the region of interest (ROI) for imaging. After imaging, the flow rate was increased 2× and the phantom was washed with at least 10 mL PBS.

### Optoacoustic imaging

A pre-clinical multi-spectral optoacoustic tomography (MSOT) device (MSOT inVision 256, iThera Medical, Munich, Germany) was used for imaging. It is equipped with an array of 256 detector elements which are cylindrically focused, having a central ultrasound frequency of 5 MHz and up to 270° coverage. The phantoms were aligned so that the illumination ring coincides with the detection plane, i.e. the curved transducer array being centered around the phantom. Data acquisition was performed in the wavelength range 680–900 nm in 10 nm steps, using 10 averages frames per wavelength, which equates to 1 s acquisition time per wavelength per section. The optical excitation originates from a Q-switched Nd:YAG laser with a pulse duration of 10 ns and a repetition of 10 Hz. Light is homogenously delivered to the phantom using a fiber split into 10 output arms. The fiber bundle and the transducer array are stationary, and the sample holder moves along the z-direction allowing longitudinal acquisition of different imaging planes using a moving stage. MSOT measurements were performed in a temperature controlled water bath at 34 °C. All of the variable parameters during the measurement were kept constant, i.e. optoacoustic gain, laser power, focus depth, frame averaging, frame rate and high/low pass filters. For optimal acoustic coupling, we waited at least 5 min before initiating the scan, so that the phantom equilibrates to the temperature of the water bath before measurement. To compare the optoacoustic signal of ICG-pHLIP and QC1-pHLIP, serial dilutions at 2 µM, 5 µM, 10 µM, 15 µM and 20 µM in DMSO were prepared. Solutions were delivered to the center of the imaging phantom device using a syringe pump. In between measurements, the flow device was washed with PBS (10 mL).

### Optoacoustic image data processing

Spatial reconstruction and multi-spectral processing (MSP) of the data was performed using the ViewMSOT software suite (V3.6; iThera Medical) and a backprojection algorithm at 200 mm (100 µm) resolution. The normalized optoacoustic reference spectra (shown in Fig. 2e) were obtained from optoacoustic phantom scans. For multi-spectral unmixing, a linear regression method was used and excluded pixels where the number of wavelengths (>25%) fell within the negative signal. Hemoglobin (HbO_2_) and deoxyhemoglobin (Hb) reference spectra are included in the software package. Orthotopic tumors were implanted in the same position and distance from the surface of the skin and from animal to animal. Hence, all of the animals are affected by light scattering to the same extent. In addition to the relative known location (mm) of the animal placed on the mice bed, the location of tumor was identified based on the presence of deoxyhemoglobin. Quantitative information was obtained by defining the ROI manually within a 2D slice MSOT image. For spectrally unmixed injected pHLIPs, the mean pixel intensities per cross-section in the volume of interest (VOI) was first plotted as classical least square (CLS) score vs. position (mm) to assess the signal strength in the tumor. Decreasing signal strength over the distance away from the tumor, creating a parabolic shape is in agreement with the analysis. The maximum “mean signal per cross-section” was used as a quantitative indicator of probe binding in the tumor. Overall optoacoustic intensities (a.u.) were reported at all imaging wavelengths (680–900 nm). For all cases, unless otherwise stated, the (CLS) method for spectral deconvolution was used and reported as CLS scores. The following reference spectra were used: hemoglobin (HbO_2_), deoxyhemoglobin (Hb) and either ICG-pHLIP or QC1-pHLIP. In order to compare the direct intensities of all the images between ICG-pHLIP and QC1-pHLIP, the optoacoustic spectra (2 µM) were normalized to their maximum optoacoustic (810 nm) wavelength. The overall optoacoustic intensities (a.u., *bone scale*) are then overlaid with either multi-spectrally unmixed ICG-pHLIP (*green*) or QC1 (*cyan*) pHLIP channel.

### Statistical analysis

Unpaired t-tests were performed to determine the statistical difference for *in vitro* experiments and between *ex vivo* non-injected and injected groups. Paired t-tests were performed to determine the statistical difference *in vivo* before and after injection. Levels of significance are as follows: ns = not significant, *p < 0.05, **p < 0.01, ***p < 0.001 and ****p < 0.0001. Data is presented as mean ± SD.

### *In vitro* fluorescence and optoacoustic imaging of ICG-pHLIP and QC1-pHLIP

A solution of cell free media containing saline, 20% DMSO and either ICG-pHLIP or QC1-pHLIP (10 µM) was prepared. A second preparation contained murine breast cancer 4T1 cells, which had been grown and maintained in Roswell Park Memorial Institute (RPMI) media (pH 7.4) and 10% fetal bovine serum (FBS) with penicillin/streptomycin under standard cell culture conditions (5% CO_2_ in air at 37 °C). At 70% confluence, the RPMI media was removed, washed with PBS, trypsinized and suspended in RPMI media. The cells were counted (1:2 dilution) and the appropriate volumes transferred into separate falcon tubes (0.1 × 10^6^, 0.5 × 10^6^, 1 × 10^6^ and 5 × 10^6^). The cells were spun down into pellets (1200 rpm for 5 min) and carefully re-suspended into the appropriate volume of saline. The solution containing the pHLIP imaging agents (35 µL) was added to the cell suspension (35 µL). The cells were mixed thoroughly by pipetting up and down. After 10 min incubation time, optoacoustic and fluorescent imaging was carried out.

### Animal studies

All animal experiments were done in accordance with protocols approved by the Institutional Animal Care and Use Committee (IACUC) of Memorial Sloan Kettering Cancer Center (MSKCC) and followed the National Institutes of Health guidelines for animal welfare. Healthy Hsd:athymic female mice NudeFoxn1^nu^ (6–8 weeks old) were used in the study. The mice were split into three groups of four mice, namely for the ICG-pHLIP injected group, the QC1-pHLIP injected group and a non-injected control group. All animal procedures, other than tail vein injections, were performed with the animals under general 2% isoflurane inhalation anesthesia. In order to test the ability of ICG-pHLIP and QC1-pHLIP to target tumor tissue, orthotopic allografts were created using the mouse breast cancer cell line 4T1. The 4T1 cells were injected into the 4^th^ mammary fat pad just inferior to the nipple of a female mouse (1 × 10^6^ cells in 30 µL 1:1 RPMI medium and Matrigel). The tumors were allowed to grow for 6–7 days (typically reaching a volume estimated from caliper measurements of 100–150 mm^3^) before the ICG-pHLIP or QC1-pHLIP formulation were injected. The mice were equally split into groups of three cohorts (n = 4): (i) An ICG-pHLIP injected group; (ii) a QC1-pHLIP injected group; (iii) and a non-injected group. Tumor-bearing mice (n = 4) were imaged with the MSOT prior to injection. The experiments were randomized, and 200 µL of 6 mM kg^−1^ of ICG-pHLIP or QC1-pHLIP formulation were injected into mice via tail vein. The mice were re-imaged at 24 h post injection. Both pHLIP agents were dissolved in 10% v/v glucose in phosphate buffer saline (PBS). For MSOT imaging timepoints, the experiments were conducted on the murine breast cancer model (n = 3) that were injected with 200 µL of 120 µM QC1-pHLIP in PBS. Tumor optoacoustic signals were monitored non-invasively using the MSOT imaging system after 30 min, 4 h, 8 h, 12 h and 24 h post intravenous injection. Animals injected with ICG-pHLIP were imaged at 30 min, 4 h, 8 h, 12 h and 24 h on an IVIS Spectrum (PerkinElmer, Waltham, MA, USA) system for planar fluorescence imaging. Perfused and non perfused mice studies shown in Fig. S8 were a group of mice where n = 3. In general, pHLIP imaging agents were allowed to circulate for 24 h to study and assess the distribution and accumulation of the contrast agent using fluorescent and optoacoustic imaging.

### *Ex vivo* imaging of tissue sections

After 24 h post intravenous injection and imaging, muscle and tumors were harvested and fixed in 4% paraformaldehyde (PFA, MP Chemicals, Solon, OH) in PBS overnight at 4 °C, thoroughly rinsed with PBS, then kept in 70% ethanol. Tissues were embedded in paraffin and 5 mm thick sections were sliced (10 µm) from the paraffin block. The sections were either imaged with confocal microscope (Leica TCS SP8) or stained with hematoxylin and eosin (H&E) and scanned with a Mirax digital slide scanner (Zeiss, Jena, Germany) for histological analysis.

## Supplementary information

Supplementary Info- optoacoustic imaging of QC1 pHLIP
