# Supplementary material for: Acid specific dark quencher QC1 pHLIP for multi-spectral optoacoustic diagnoses of breast cancer
Source: Sci Rep. 2019 Jun 12;9:8550. doi: 10.1038/s41598-019-44873-1 (PMC6561946; doi:10.1038/s41598-019-44873-1)
Supplement: Supplementary file 1 — Supplementary Info- optoacoustic imaging of QC1 pHLIP [file 41598_2019_44873_MOESM1_ESM.pdf]

Supplementary Information for

Acid specific dark quencher QC1 pHLIP for multi-spectral optoacoustic diagnoses of breast cancer

Sheryl Roberts[1], Arianna Strome[1], Crystal Choi[1], Chrysafis Andreou[1], Susanne Kossatz[1], Christian Brand[1], Travis Williams[1], Michelle Bradbury[1,2], Moritz F. Kircher[1,2,3,4,5], Yana K. Reshetnyak[6], Jan Grimm[1,4,7,8], Jason S. Lewis[1,4,8,9,10], Thomas Reiner*[1,4,11]

[1]Department of Radiology, Memorial Sloan Kettering Cancer Center, 1275 York Avenue, New York, New York, 10065, USA

[2]Molecular Pharmacology Program, Sloan Kettering Institute for Cancer Research, New York, New York 10065, USA

[3]Center for Molecular Imaging and Nanotechnology (CMINT), Memorial Sloan Kettering Cancer Center, New York, NY, 10065, USA

[4]Department of Radiology, Weill Cornell Medical College, 1300 York Avenue, New York, New York, 10065, USA

[5]Department of Imaging, Dana-Farber Cancer Institute/Harvard Medical School, Boston, MA 02215, USA

[6]Department of Physics, University of Rhode Island, 2 Lippitt Rd, Kingston, RI 02881, USA

[7]Department of Molecular Pharmacology, Memorial Sloan Kettering Cancer Center, New York, New York, USA

[8]Department of Pharmacology, Weill Cornell Medical College, New York, NY, USA

[9]Molecular Pharmacology Program, Memorial Sloan Kettering Cancer Center, New York, NY, USA

[10]Radiochemistry and Molecular Imaging Probes Core, Memorial Sloan Kettering Cancer Center, New York, NY, USA

[11]Chemical Biology Program, Memorial Sloan Kettering Cancer Center, New York City, NY 10065, United States

* corresponding author: reinert@mskcc.org, 646-888-3461

a

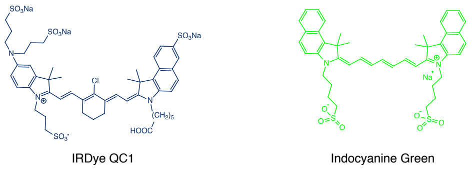

b

name	amino acid sequence	Mw
pHLIP	(D-Ala)-(D-Cys)-(D-Asp)-(D-Asp)-(D-Gln)-(D-Asn)-(D-Pro)-(D-Trp)-(D-Arg)-(D-Ala)-(D-Tyr)-(D-Leu)(D-Asp)-(D-Leu)-(D-Leu)-(D-Phe)-(D-Pro)-(D-Thr)-(D-Asp)-(D-Thr)-(D-Leu)-(D-Leu)-(D-Leu)-(D-Asp)-(D-Leu)-(D-Leu)-(D-Trp)	3221.67
ICG pHLIP	(D-Ala)-(D-Cys-maleimido-mono-amide-ICG)-(D-Asp)-(D-Asp)-(D-Gln)-(D-Asn)-(D-Pro)-(D-Trp)-(D-Arg)-(D-Ala)-(D-Tyr)-(D-Leu)-(D-Asp)-(D-Leu)-(D-Leu)-(D-Phe)-(D-Pro)-(D-Thr)-(D-Asp)-(D-Thr)-(D-Leu)-(D-Leu)-(D-Leu)-(D-Asp)-(D-Leu)-(D-Leu)-(D-Trp)	4148.53
QC1 pHLIP	(D-Ala)-(D-Cys-maleimido-mono-amide-QC1)-(D-Asp)-(D-Asp)-(D-Gln)-(D-Asn)-(D-Pro)-(D-Trp)-(D-Arg)-(D-Ala)-(D-Tyr)-(D-Leu)-(D-Asp)-(D-Leu)-(D-Leu)-(D-Phe)-(D-Pro)-(D-Thr)-(D-Asp)-(D-Thr)-(D-Leu)-(D-Leu)-(D-Leu)-(D-Asp)-(D-Leu)-(D-Leu)-(D-Trp)	4348.43

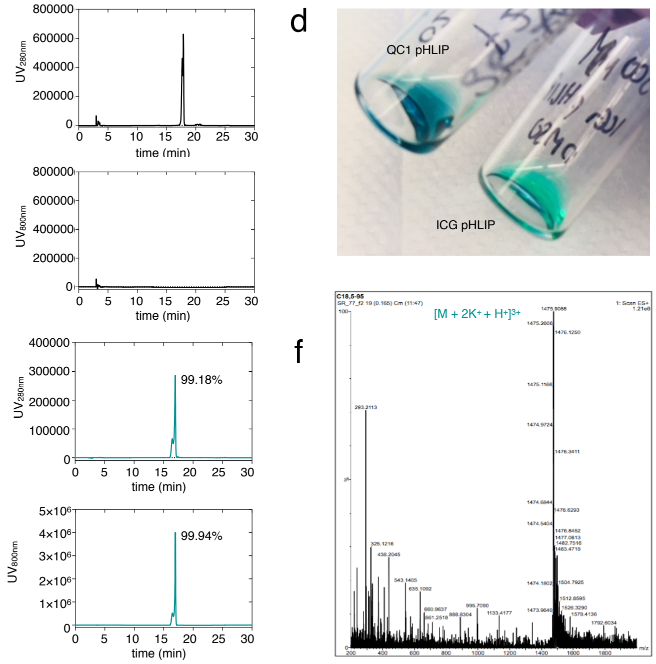

Fig. S1. Chemical characterizations of sonophore QC1 pHLIP. (a) Chemical structure of IRDye QC1 (*left*) and indocyanine green (ICG, *right*). (b) Amino acid sequence and molecular weight of var3 pHLIP, ICG pHLIP and QC1 pHLIP (c) HPLC chromatogram of var3 pHLIP at 280 nm (*top*) and 800 nm (*bottom*). (d) The appearance of ICG pHLIP and QC1 pHLIP at the same concentration in DMSO. (e) HPLC chromatogram of QC1 pHLIP at 280 nm (*top*) and 800 nm (*bottom*). (f) LC-MS of QC1 pHLIP ESI$^+$.

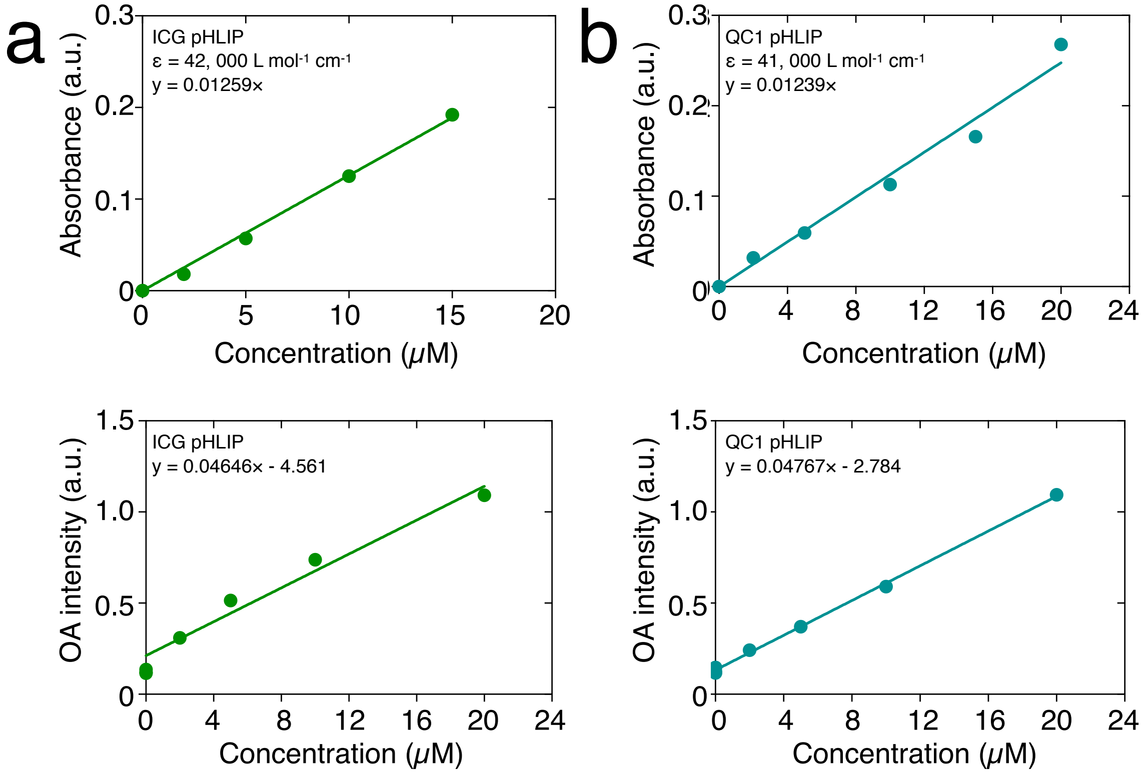

Fig. S2. Calibration curves plotted from the UV/Vis absorbance spectra and optoacoustic spectra of ICG pHLIP (a) and QC1 pHLIP (b) sonophores. The UV/Vis absorbance (*top*) and optoacoustic intensities (*bottom*) were measured in phosphate buffer saline (PBS), pH 7.20 containing 10 mM D-glucose at varying concentrations of sonophores (2 μM, 5 μM, 10 μM, 15 μM and 20 μM) as indicated on the graph. The extinction coefficient (ε) was determined using the slope calculated at λ_{max} at 810 nm.

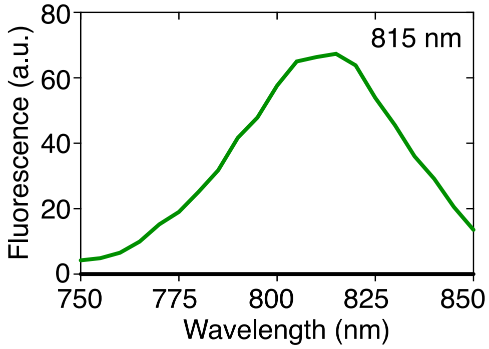

Fig. S3. The fluorescent spectra of ICG pHLIP in methanol. It has an emission maximum at 815 nm (excited at 660 nm and using a 695 cutoff filter). The solution was 7 μM.

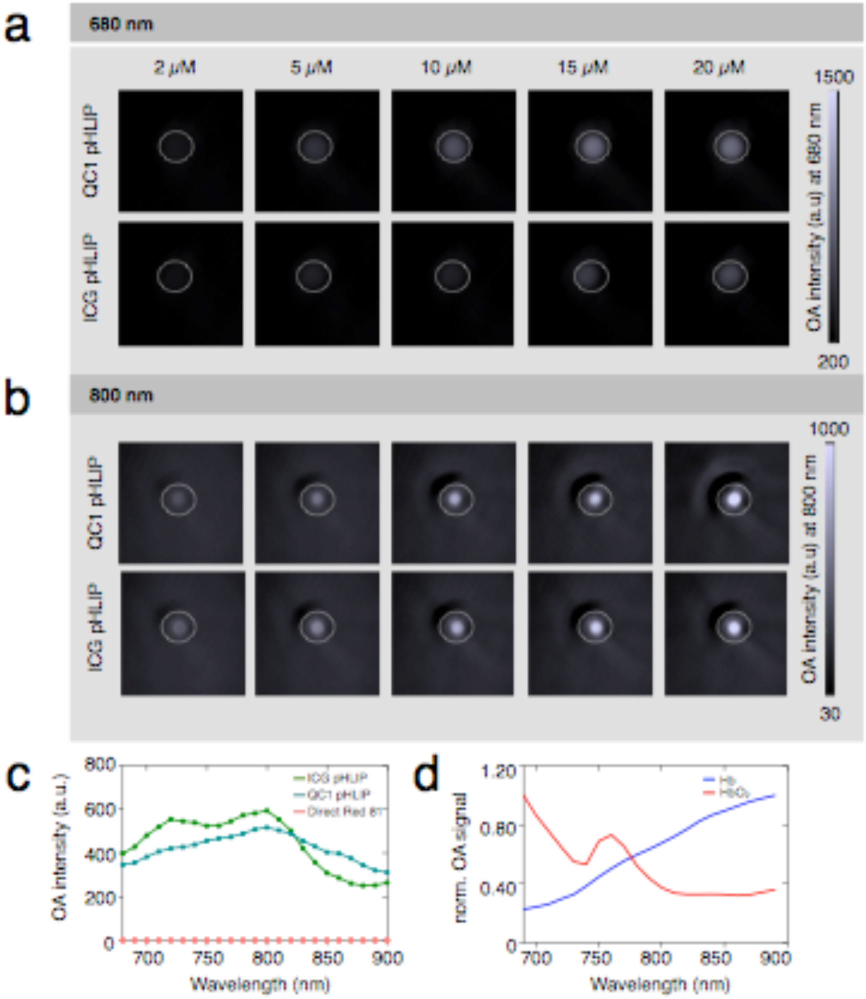

Fig. S4. Direct red 81 has little or no effect on spectral coloring due to light fluence. The optoacoustic intensity images (a.u.) of ICG pHLIP and QC1 pHLIP at 680 nm (a) and 800 nm (b) with increasing concentrations of 2 μM, 5 μM, 10 μM, 15 μM and 20 μM (*left to right*) and (c) Optoacoustic intensities (a.u.) of ICG pHLIP, QC1 pHLIP and Direct Red at 5 μM. (d) Normalized spectra of deoxy haemoglobin (Hb) and haemoglobin (HbO_2); spectra included as references for multi-spectral unmixing analysis.

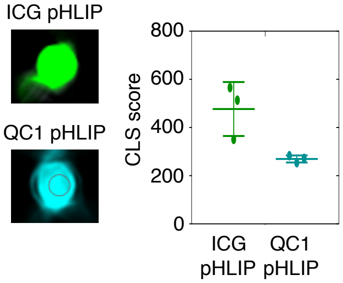

Fig. S5. Optoacoustic imaging of ICG pHLIP and QC1 pHLIP at the same concentration of 5 μM in phosphate buffer saline (PBS). The mean optoacoustic intensities (a.u.) of ICG pHLIP is 477 ± 112 and QC1 pHLIP is 245 ± 9 (n = 3). The result is comparable to the one carried out in DMSO at 5 μM.

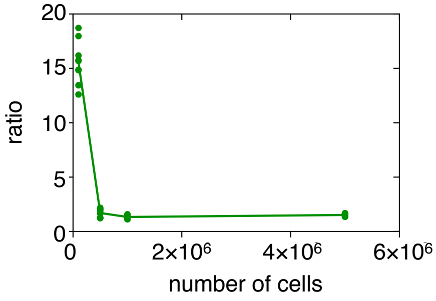

Fig. S6. Fluorescence of ICG pHLIP in the absence and presence of cells. *In vitro* fluorescence using increasing amounts (100 000 cells, 500 000 cells, 1 000 000 cells and 5 000 000 cells) of the murine breast cancer cell line 4T1 were incubated with 5 µM ICG pHLIP. No washing steps were carried out. Data was collected in biological triplicates of triplicates. The mean at each group was calculated (*n* = *9*). The ratio difference was calculated by taking the fluorescence and dividing by the mean of the previous group. ICG pHLIP fluorescence increases roughly 16-fold in presence of cells.

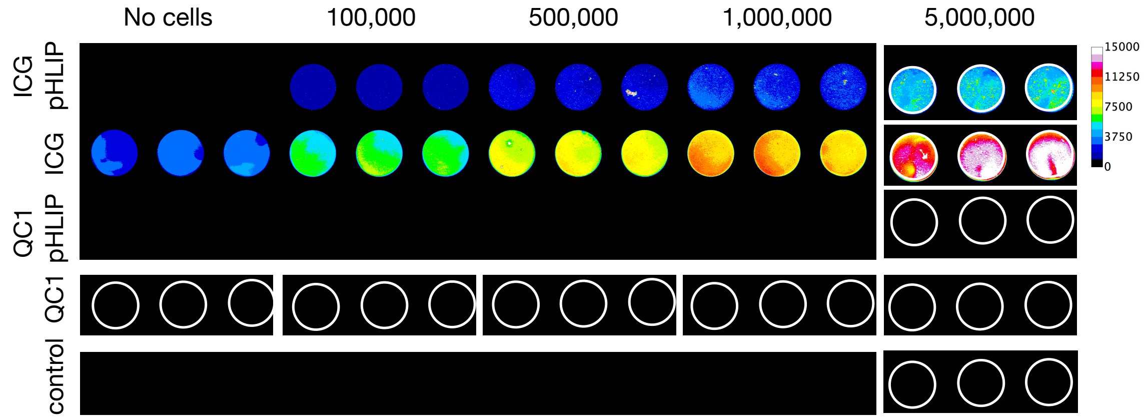

Fig. S7. Membrane insertion into the tumor turns on fluorescence of ICG pHLIP increases as we increase concentrations of 4T1 cells. *In vitro* fluorescent imaging of ICG pHLIP, ICG, QC1 pHLIP and IRDye QC1 at 5 μM and carried out at varying concentrations of 4T1 cells prepared from 0 cells, 100 000 cells, 500 000 cells, 1 000 000 cells and 5 000 000 cells. The cells were then incubated with 5 μM of either ICG pHLIP or QC1 pHLIP for 10 min at room temperature. The images were obtained from the Odyssey scanner at 800 nm. See corresponding quantification in Fig. 2b.

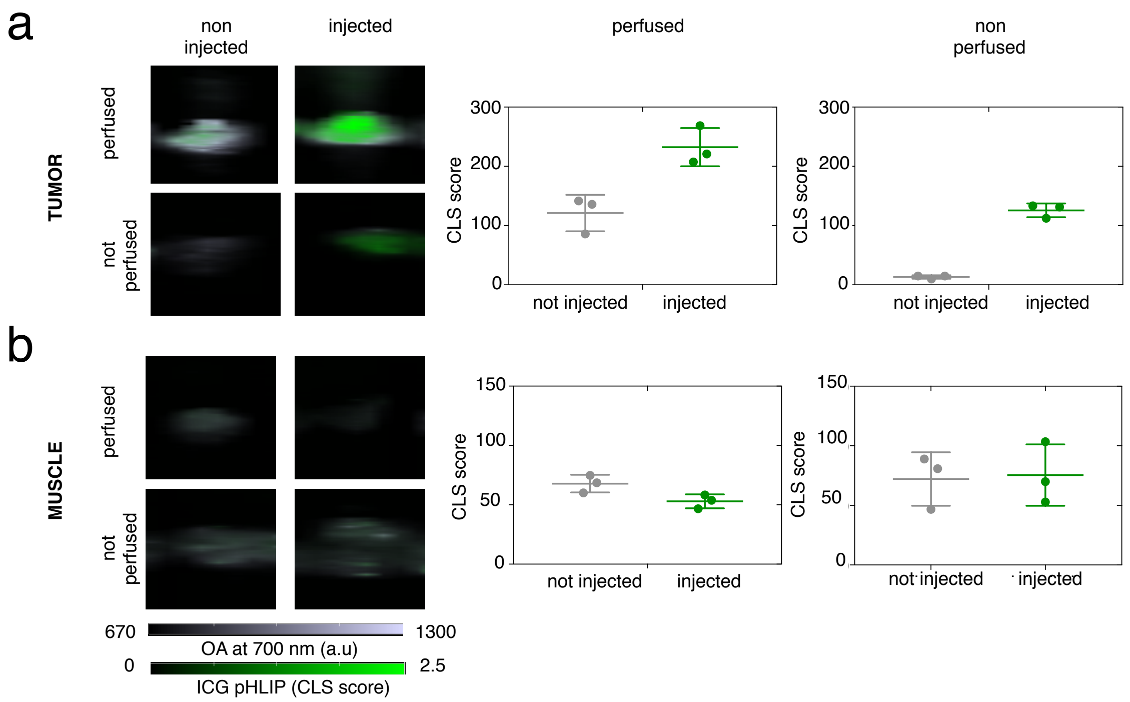

Fig S8. Perfusion for *ex vivo* imaging improves signal to noise (S/N) in optoacoustic tomography. Using orthotopic murine breast cancer model and injecting 120 μM of ICG pHLIP, representative images of optoacoustic signals are shown for non injected (*left*) and injected (*right*) tumors (a) and muscles (b) that are either perfused (*top*) or not perfused (*bottom*).

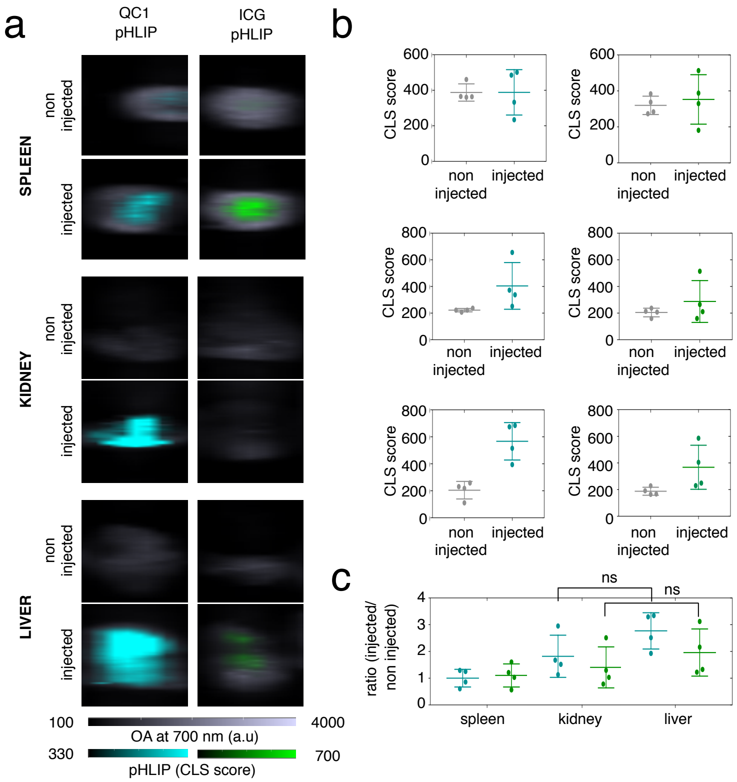

Fig. S9. Dark quencher QC1 pHLIP outperforms ICG pHLIP in *ex vivo* optoacoustc imaging of spleen, kidney and liver at 24h post intravenous injection in orthotopic murine breast cancer model. (a) Representative images showing that QC1 pHLIP can still delineate better than ICG pHLIP even at organs (spleen (*top*), kidney (*middle*) and liver(*bottom*)) that contain high concentrations of blood (non injected $n = 4$, injected $n = 4$). (b) Respective optoacoustic quantification of spleen (*top*), kidney (*middle*) and liver (*bottom*) between QC1 pHLIP (*left*) and ICG pHLIP (*right*). (c) Signal ratio between the injected over non injected respective organs of QC1 pHLIP (*cyan*) and ICG pHLIP (*green*). Ns = not significant, *p<0.05, **p<0.01, ***p<0.001, ****p<0.0001 (unpaired t-test).

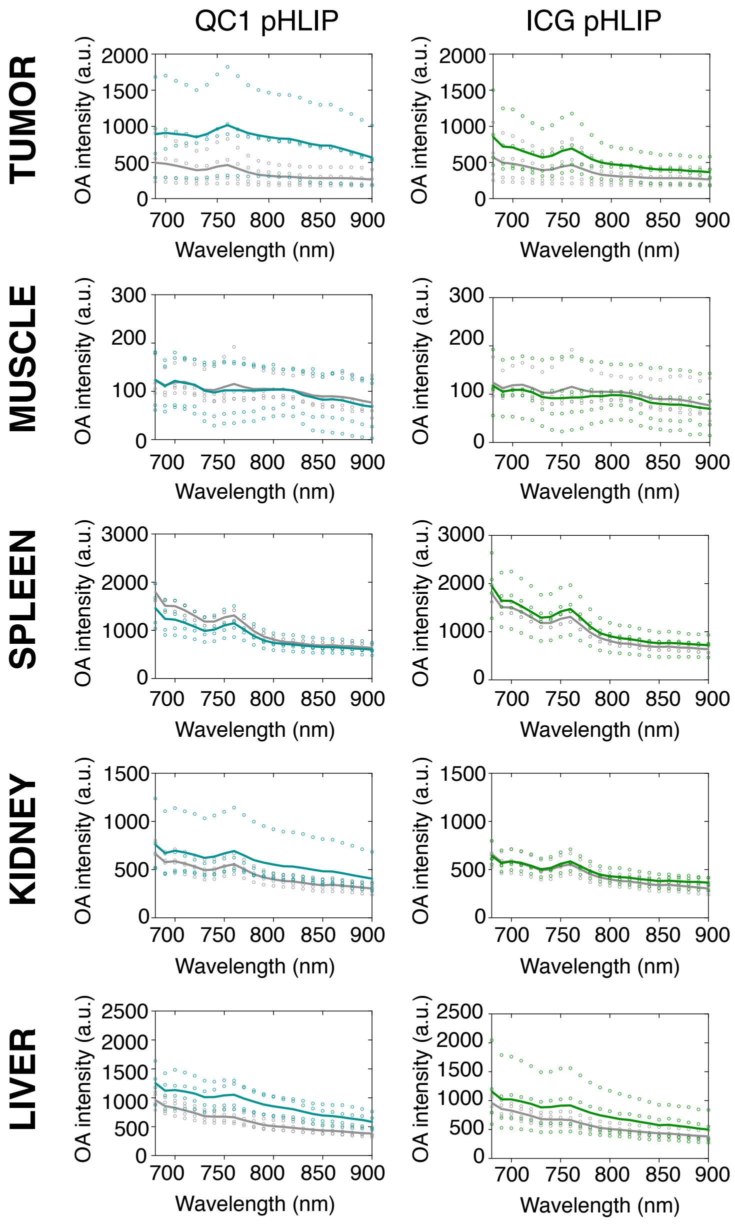

Fig. S10. The overall *ex vivo* optoacoustic intensities between the injected and non injected organs. Optoacoustic intensities (a.u.) of tumor, muscle, spleen, kidney and liver (*top to bottom*) are shown between QC1 pHLIP (*cyan*) and ICG pHLIP (*green*) against non injected controls (*grey*).

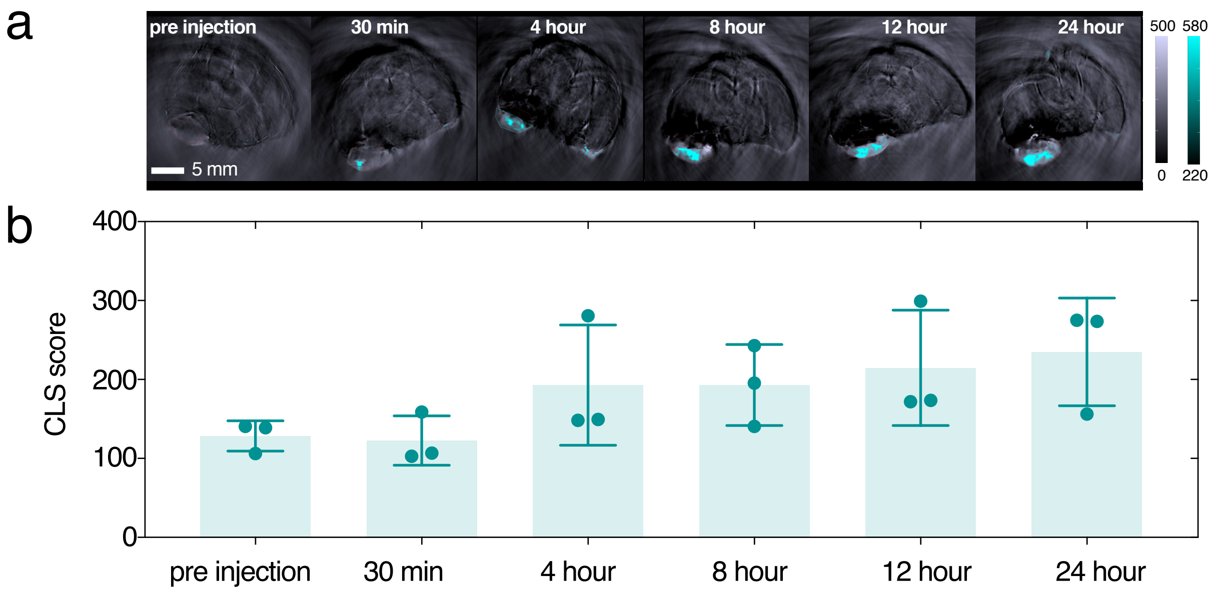

Fig. S11. The *in vivo* accumulation of QC1 pHLIP over time using optoacoustic imaging. (a) Top images are representative of *in vivo* optoacoustic imaging of QC1 pHLIP taken at various timepoints before and after intravenous injection of QC1 pHLIP at 30 min, 4 h, 8 h, 12 h and 24 h (*left to right*). (b) The corresponding relative optoacoustic signal increase of QC1 pHLIP over time.

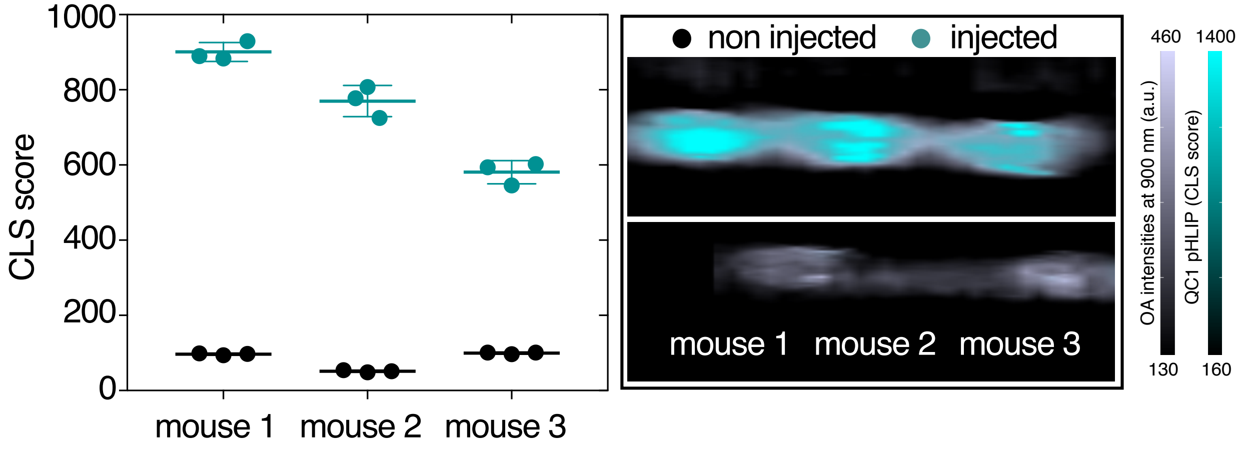

Fig. S12. Mice were intravenously injected with dark quencher QC1 pHLIP (120 μM), allowed 24h accumulation of the probe before its tumors were excised out (n = 3) and compared with the non injected control (n = 3). Note that these ex vivo injected tumors corresponds to the mice that were imaged in vivo at various timepoints (Fig. S11).

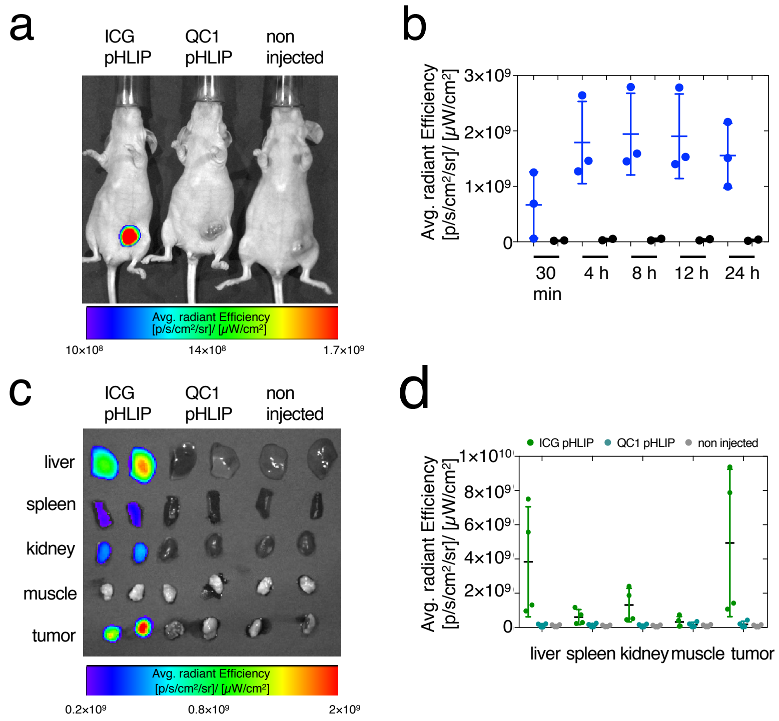

Fig. S13. *In vivo* and *ex vivo* kinetics and biodistribution validation of pHLIPs using fluorescence imaging. (a) Representative *in vivo* fluorescent images of ICG pHLIP (*left*), QC1 pHLIP (*middle*) and non injected (*right*) orthotopic murine breast cancer model (*n* = *3*) at 24h post injection at 120 μM of agent. (b) Quantification of the *in vivo* tumor region of interest (ROI) from the fluorescent images acquired at 30 min, 4h, 8h, 12h and 24h post injection (blue = injected, black = non injected). (c) Representative *ex vivo* fluorescent images of liver, spleen, kidney, muscle and tumor (*top to bottom*) tissues excised out at 24h post *i.v.* injection of pHLIPs. (d) Quantification of the *ex vivo* organs by drawing ROI from the fluorescent images.

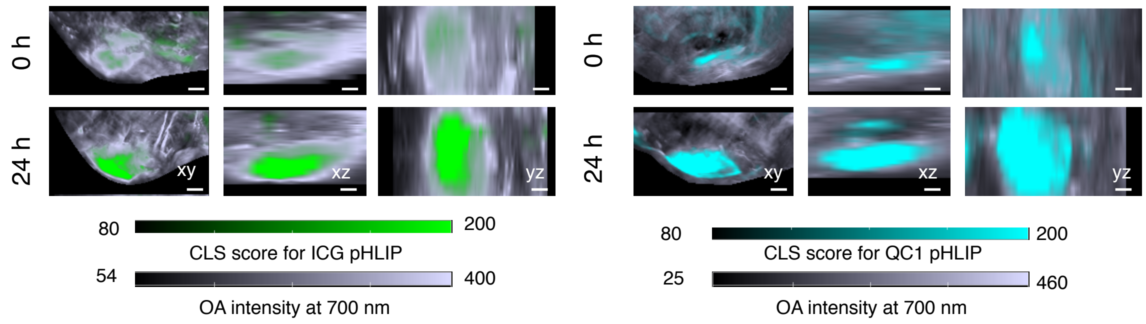

Fig. S14. The maximum intensity projections (MIP) of tomographic sections of tumors from three different 2D planes (*left to right* xy, xz and yz) demonstrates the overall targeted distribution of both pHLIPs through the tumor. Scale bar (white) is 2.5 mm.

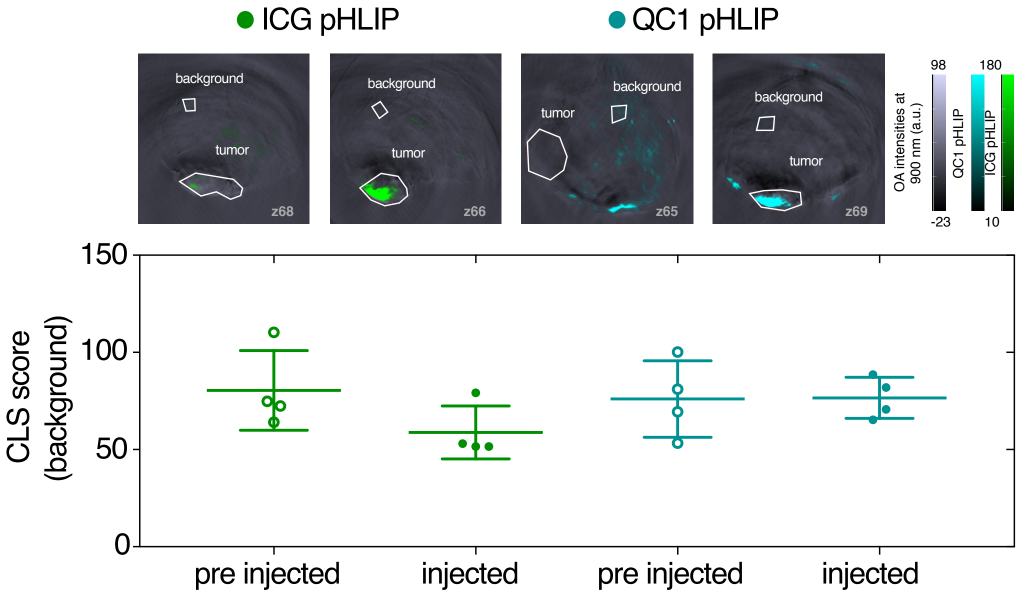

Fig. S15. The optoacoustic background before and 24 h post intravenous injection of pHLIP. Representative region of interests (ROI) for background and tumor are marked (white).

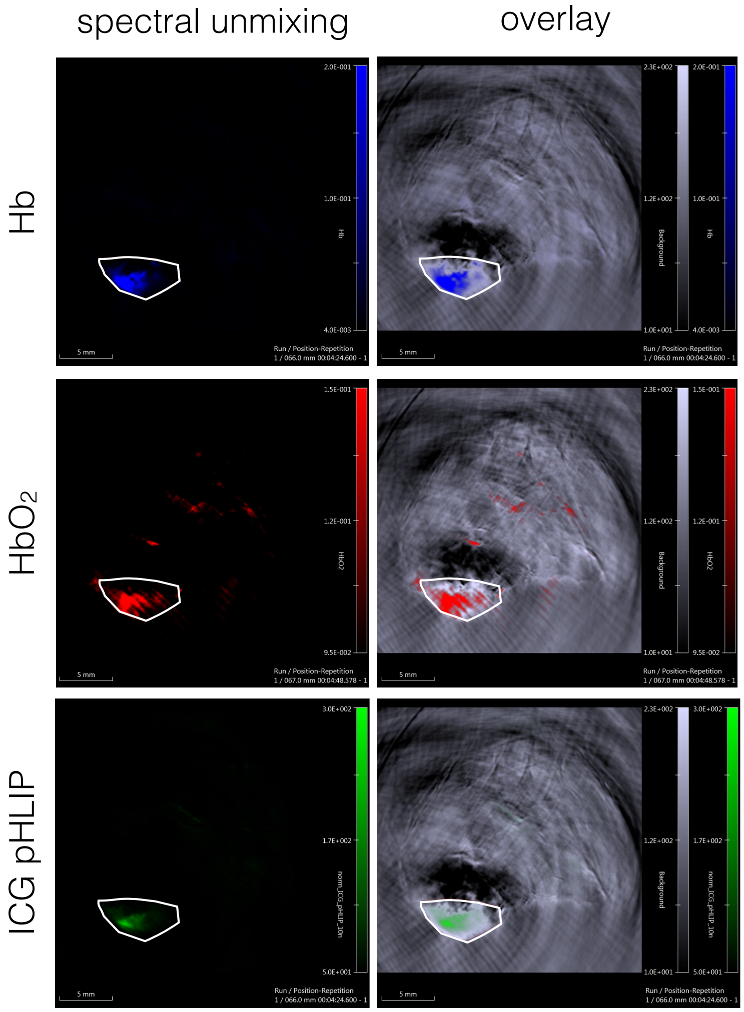

Fig. S16. Determining tumor margins using spectrally unmixed oxy/deoxyhemoglobin channel. Representative images at 24 h post intravenous injection of ICG pHLIP showing the three split channels for deoxyhemoglobin (Hb, *top*), hemoglobin (HbO_2, *middle*) and ICG pHLIP (*bottom*).

--------------- end of document -------------
